# The Complement System and C1q in Chronic Hepatitis C Virus Infection and Mixed Cryoglobulinemia

**DOI:** 10.3389/fimmu.2018.01001

**Published:** 2018-05-29

**Authors:** Ahmed El-Shamy, Andrea D. Branch, Thomas D. Schiano, Peter D. Gorevic

**Affiliations:** ^1^Division of Liver Disease, Department of Medicine, Icahn School of Medicine at Mount Sinai, New York, NY, United States; ^2^Department of Pharmaceutical and Biological Sciences, California Northstate University, Elk Grove, CA, United States; ^3^Division of Rheumatology, Department of Medicine, Icahn School of Medicine at Mount Sinai, New York, NY, United States

**Keywords:** liver, hepatitis C virus, complement, C1q, gC1qR, mixed cryoglobulinemia

## Abstract

The complement system bridges innate and adaptive immunity against microbial infections, with viral infection being a major trigger. Activation of the classical, alternative, and lectin pathways have been reported in chronic hepatitis C virus (HCV) infection and/or cryoglobulinemia. HCV infection leads to dysregulation of complement-mediated immune responses. Clinical and experimental evidence support involvement of complement in intra- and extrahepatic manifestations of HCV infection, such as liver fibrosis and type II cryoglobulinemia. In this review, we summarize studies that have investigated the interplay between HCV and the complement system to establish chronic infection and autoimmunity, as well as the association between HCV pathogenesis and abnormal complement profiles. Several unanswered questions are highlighted which suggest additional informative lines of investigation.

## Introduction

The complement system includes major host defense mechanisms that bridge innate and adaptive immunity against microbial infections. It is also a critical mediator of the clearance of immune complexes and injured cells (1–3). Dysregulation may be associated with chronic autoimmune inflammatory conditions, such as systemic lupus erythematosus, cryoglobulinemia, and rheumatoid arthritis. Viral infections may trigger dysregulation by direct effects on complement components for the purpose of immune evasion, by effects on specific receptors used for viral entrance into cells or by promoting a pathogenic antiglobulin [i.e., rheumatoid factor (RF)] response as part of chronic immune stimulation. In particular, hepatitis C virus (HCV) infection has been associated with a number of extrahepatic disorders, such as type II mixed cryoglobulinemia (MC) and B cell lymphoma that may be accompanied by complement dysregulation. The recent introduction of direct-acting antiviral (DAA) therapy for HCV allows most patients to achieve a sustained virological response (SVR)/cure. Treatment reduces liver inflammation and improves extrahepatic disease manifestations, but cryoglobulinemia and liver dysfunction may persist (4–14).

The majority of HCV-infected patients with evidence of B cell clonality have abnormal complement profiles; a low serum level of C4 is a “signature” for type II MC patients (15, 16). However, only limited information is available regarding the mechanisms by which the complement system is involved in HCV-induced intra- and extrahepatic disease. Therefore, in this review, we aim to highlight the interplay between HCV and the complement system that become apparent with chronic infection and lymphoproliferation.

## Production Sites of Complement Proteins and Receptors

More than 40 complement-related proteins have been identified in the plasma and on cell surfaces, constituting more than 15% of the of plasma globulins (17, 18). The liver makes ~90% of the plasma components of classical, alternative, and lectin pathways. By contrast, whereas hepatocytes are the predominant source of C1r and C1s in blood, activated monocytes/macrophages and immature dendritic cells are the primary source of C1q, a recognition molecule for classical pathway activation that also has significant non-complement functions (19). Although the liver produces the majority of C4, multiple tissues may also produce this protein for local consumption, particularly in response to interferon-gamma (20). Several complement receptors, such as complement receptor type 1 (CR1), the complement receptor of the immunoglobulin superfamily (CRIg), the integrin complement receptors 3 (CR3) and 4 (CR4), and the complement component 5a receptor 1 (C5aR), are expressed on liver cells (hepatocytes, endothelial cells, Kupffer cells, and stellate cells), and contribute to a variety of functions, such as induction of gluconeogenesis, synthesis of acute-phase proteins, hepatocyte proliferation, and phagocytosis (21).

## Fibrosis and Regeneration

Increasing evidence implicates activation of the complement system in the pathogenesis and response to acute and chronic liver injury (22). In a clinical study investigating the relationship between complement activation and development of liver fibrosis, blood samples from 50 chronically infected HCV patients were compared to 35 patients with other various liver diseases and 50 healthy controls (23). Complement system activation, as indicated by a significant decrease in total plasma complement activity (CH50) and increase in SC5b-9 [a marker for generation of the membrane-attack complex (MAC)] was associated with high necroinflammatory activity in the HCV patients and patients with other liver diseases compared to controls. The level of SC5b-9 significantly correlated with liver fibrosis stage in HCV-infected patients but not in patients with other liver injuries. In a proteomic survey of serum samples from HCV-infected patients, liver fibrosis stage was associated with a decrease in C3, C4, and Factor H-related protein-1, a regulatory C3b-binding protein (24). C4a, a cleavage product of C4 that contrasts with C3a and C5a with regard to biologic function, was reported to be negatively correlated with the stage of liver fibrosis in children with chronic HCV infection, serum levels being significantly lower in HCV children with advanced fibrosis than those with no/mild fibrosis (25); the significance of this study, however, was limited by the low numbers of HCV-infected children, and the fact that C4a may increase as a function of classical pathway activation and C4 consumption. In a murine genomic study, the gene encoding C5 was identified as a quantitative trait locus associated with the development of liver fibrosis (26). Expression of C5aR1 was significantly upregulated on hepatic stellate cells during trans-differentiation to myofibroblasts in culture. Since myofibroblasts synthesize collagens and other extracellular matrix proteins, elevated C5aR1 expression is consistent with the concept that C5 is a modifier of liver fibrogenesis. Indeed, blockade of C5aR1 reduces hepatic fibrosis in mice (26). Critical roles for C3a and C5a for liver regeneration following carbon tetrachloride injury have been shown in C3, C5, and C3aR knock-out and double knock-out mice, reversible by restoration of C3a, C5, or C5a (27). Interestingly, in 277 patients with chronic HCV infection, two C5 single-nucleotide polymorphisms (SNPs), rs17611 and rs2300929, were associated with advanced fibrosis (26). However, in a study of 1,435 HCV-infected patients and 1,003 patients with other liver diseases, there was no significant association between these C5 SNPs and fibrosis in either patient group (28).

## Hepatocellular Carcinoma (HCC)

Cancer growth is determined by intrinsic properties of malignant cells as well as several modifiers, including the complement system, which may either reduce or increase progression (29). HCC is the second leading cause of organ-specific cancer-related death worldwide (30) and is the most rapidly increasing cause of cancer-related death in the United States, Europe, and Japan (31). Of note, C3a was identified by mass spectrometry and 2-dimensional gel electrophoresis (2-DE) of serum samples obtained from HCV-HCC, chronic HCV, HBV-HCC, chronic HBV, and healthy subjects as a differentially expressed biomarker protein with significantly higher levels among HCV-HCC patients compared to the other groups (32).

## Treatment

Until recently, HCV treatment has centered on interferon-alpha (IFNα), a cytokine with both antiviral and immunologic effects. However, IFNα-based treatment failed to eliminate HCV in many patients and was often poorly tolerated, particularly because of its ability to induce, uncover and/or exacerbate autoimmune/inflammatory disorders (33, 34). The CC genotype associated with the rs285009 SNP of the C4 gene closely correlated with decreased level of mRNA expression and C4 protein which was more striking at baseline in HCV patients compared to healthy controls. More importantly, the presence of this SNP was significantly associated with a poor response to IFNα-based therapy as well as the development of a high degree of liver fibrosis (35). Interestingly, a significant reduction in C4 activity was also observed in relapsers after IFNα treatment compared to patients who achieved SVR (36). Polymorphism at the rs2230201 SNP of C3 was also associated with IFNα treatment outcome (37). The rs2230201 ‘C’ allele was associated with increased serum C3 levels compared to the ‘T’ allele, which conferred an advantage in attaining SVR, especially in homozygotes. Patients with serum C3 value < 53 mg/dL and non-CC genotypes may not respond to IFNα treatment (37). Recently developed DAA therapies provide an opportunity for HCV patients with autoimmune/inflammatory disorders to be cured with a low risk of side effects (38–40). In the era of DAA, how a patient’s complement profile contributes to the treatment response remains to be defined.

In summary, accumulating clinical observations support a role for the complement system in mediating liver inflammation and fibrosis in HCV infection (Table 1). However, the mechanisms underlying these observations are still unclear. Thus, further experimental and molecular studies are required to dissect how the complement system contributes to intrahepatic HCV pathogenesis, including roles in innate and adaptive immunity, regulation of apoptosis, fibrosis, and regeneration.

**Table 1:** Complement system abnormalities in HCV-induced liver injury.

Complement abnormality	HCV-induced liver injury	Reference
Low plasma CH50	High necroinflammatory activity	(23)
High SC5b-9	High stage of necroinflammation and degree of liver fibrosis	(23)
Low C3	High degree of liver fibrosis	(24)
Low C4	High degree of liver fibrosis	(24, 25)
CC genotype of rs285009 single nucleotide polymorphism (SNP) of the C4 gene	High degree of liver fibrosis and poor response to IFNα-based therapy	(35)
Low factor H-related protein-1	High degree of liver fibrosis	(24)
High C5	High degree of liver fibrosis	(26)
High C3a	High risk of hepatocellular carcinoma	(32)
Low serum C3 and non-CC genotype of rs2230201 SNP of the C3 gene	Poor response to IFNα-based therapy	(37)

## HCV Strategies to Overcome Antiviral Responses of the Complement System

Hepatitis C virus lacks a DNA intermediate; thus, it is incapable of integrating into host chromosomal DNA. Despite this, unlike most RNA viruses, chronic infection is established in ~80% of cases (41) through multiple strategies to evade innate and adaptive antiviral responses (42). In part, this is accomplished directly by inhibition of complement components and/or indirectly by induction of regulators of complement activation (RCA) (Table 2). Mazumdar et al. examined the relationship between HCV infection and C3 concentrations in blood. C3 has a central role in modulating all three pathways of the complement system. In matched serum and liver biopsy samples from HCV patients, both the levels of C3 in serum and the expression of mRNA in biopsies were significantly lower compared to serum and tissue obtained from healthy donors (43). Further *in vitro* studies showed that HCV-NS5A protein strongly downregulated C3 promoter activity at the basal level. In addition, expression of the transcription factor C/EBP-β, which induces C3 promoter activity, was reduced in immortalized human hepatocytes and human hepatoma cells (Huh7) that were either infected with cell culture-adapted HCV or transfected with HCV-NS5A (43). Moreover, HCV inhibited C3 convertase activity, which is critical in promoting the activity of classical and lectin pathways of complement system (44). Infection of a hepatoma cell line with HCV resulted in inhibition of C2 expression and hence impairment of C3 convertase function. On the other hand, C3b deposition onto bacterial membrane was reduced by sera from HCV patients as compared to healthy controls, which further indicates impaired C3 convertase (44). C4 contributes to the eradication of several viral infections by its role as opsonin and by its central role in promoting the activity of the classical and lectin pathways (3). Notably, C4 protein levels in the serum and mRNA expression levels in liver tissue were lower in HCV patients compared to patients with unrelated liver diseases (45). *In vitro* studies showed that the expression levels of the two C4 isoforms (C4A and C4B) were significantly reduced in hepatocytes transfected with a full-length HCV genome. In particular, among different HCV proteins, only core and NS5A contributed to HCV’s inhibitory effect on C4 as shown by *in vitro* transfection experiments, using the Huh7 hepatoma cell line and plasmids containing different HCV proteins (45). Consistent with these *in vitro* results, the expression levels of C4 mRNA in liver tissue of HCV-core or NS5A transgenic mice were also significantly reduced. Mechanistic studies showed that HCV-core downregulated the expression of upstream stimulatory factor-1, a transcription factor critical for C4 expression, while NS5A inhibited the expression of interferon regulatory factor-1, which is required for IFN-γ-induced C4 promoter activation (45).

**Table 2:** HCV strategies to evade innate and adaptive immunity using complement system-related components.

Complement system-related factor	HCV-induced immune evasion	Reference
C3	Downregulation of C3 promotor activity by HCV-NS5A *via* inhibition of C/EBP-β	(43)
C2	Impairment of C3 convertase function *via* inhibition of C2	(44)
C4	Inhibition of C4 activity through HCV core-induced inhibition of upstream stimulatory factor-1 and HCV-NS5A-induced inhibition of interferon regulatory factor-1	(45)
C9	Impairment of membrane-attack complex (MAC) formation through inhibition of C9 promotor activity by HCV-core	(46)
C3 and C4	Downregulation of C3 and C4 hepatocyte synthesis through the inhibition of the hepatocyte MICA/B	(47)
MAC	Impairment of MAC formation through incorporation of CD59 in HCV envelope	(55)
C3 convertase	Upregulation of CD55 expression which accelerates the decay of C3 convertase	(56)
gC1qR	Impairment of T-cell immunity through the interaction of HCV core to gC1qR on T-cells and monocyte-derived dendritic cells	(65–67)

Likewise, Kim et al., showed that liver biopsies from HCV patients had lower expression of C9 mRNA compared to samples from unrelated diseases or healthy controls. This indicates that HCV regulates the MAC *via* C9. C9 mRNA was significantly downregulated in cultured hepatocytes infected with HCV (46). In particular, HCV-core protein had a critical role in regulating C9 promoter activity. Furthermore, in a subsequent *in vitro* study, HCV NS2 and NS5B proteins were found to be responsible for HCV-associated inhibition of the hepatocyte protein major histocompatibility complex class I-related chains A and B (MICA/B) which functions as a key receptor ligand for NKG2D on NK cells resulting in downregulation of C3 and C4 hepatocyte synthesis (47).

A general role for lectins and pattern recognition of viral glycoproteins has been identified for HIV and HCV (48). *In vitro* studies showed that mannan-binding lectin (MBL) bound to the HCV E2 ectodomain and E1/E2 heterodimers through its lectin domain, as well as activate complement through MBL-associated serine protease 2 (49). Ficolin-2, a known lectin pathway activator was found to inhibit attachment of HCV envelope E1 and E2 N-glycans to their low-density lipoprotein (LDL) and scavenger B1 receptors (50), with elevated blood levels of L-ficolin or MBL being found in the serum of some patients, possibly correlating with MBL2 genetic variants and response to IFNα (51).

Host expression of RCAs, such as CD35, CD46, CD55, and CD59, serves to protect the cells from MAC lysis (52, 53). HCV has developed strategies to attenuate complement activation by regulating RCAs. Amet et al. first showed that CD59, a key member of RCA, associated with the external membrane of HCV particles obtained from infected patients and Huh7.5.1 cells and had a direct role in abrogating antibody-dependent complement-mediated lysis (54). *In vitro* studies by Ejaz et al. indicated that HCV selectively incorporates CD59 in its envelope, which inhibits the formation of the MAC complex (55). Also, it was found that HCV infection upregulates the expression of CD55, which accelerates the decay of C3 convertase (56). Taken together, HCV has the capability to attenuate the complement system at multiple steps to weaken the innate immune response.

## Role of gC1q Receptor (gC1qR) in HCV Pathogenesis

gC1q receptor is an acidic multifunctional cellular protein ubiquitously expressed on somatic cells (57). It binds to the globular heads of C1q and modulates complement activation (58). Apart from its interaction with C1q, gC1qR binds to several host cell-surface ligands, such as vitronectin and high molecular weight kininogen (59). Interaction of these ligands with gC1qR leads to classical complement pathway activation with generation of inflammatory cytokines, cell adhesion, and activation of the intrinsic coagulation cascade leading to the production of bradykinin, increased vascular permeability, and infiltration of vascular tissue with proinflammatory cells (60). In addition to cellular proteins, gC1qR interacts with several microbial proteins, such as adenovirus core protein (61), HIV rev (62), and protein A of *Staphylococcus aureus* (63), suggesting its role in the pathogenesis of these infections. Interestingly, by using HCV-core protein as bait in yeast two-hybrid assay, Kittlesen et al. was the first to report the interaction between gC1qR and HCV-core (64). The interaction of HCV-core to gC1qR on T-lymphocytes resulted in inhibition of T-cell proliferation and function through impairment of ERK/MEK phosphorylation (65) and Lck/Akt activation (66). Also, engagement of gC1qR on monocyte-derived dendritic cells with HCV-core resulted in an impaired capacity to generate type 1 CD4^+^ T cell immunity *via* inhibition of TLR-induced IL-12 production (67). Therefore, HCV might utilize the direct interaction of its core protein with gC1qR on T cells as a tool to suppress cellular immunity which might imply an important role in persistent infection, an observation that might extend to minicore isoforms of this protein, which lack the RNA binding domain of the p21 core (68).

By contrast to the inhibitory influence of HCV-core protein and gC1qR interaction on T cell responses, this interaction on B cells resulted in hyper-activation and proliferation indicated by upregulation of CD69, overexpression of costimulatory and chemokine receptors, and increased production of IgM and IgG (69). This might partially explain the link between chronic HCV infection, B-cell lymphoproliferative disorders, and several autoimmune-related diseases (70–72). In support of this, the level of circulating gC1qR and gC1qR mRNA of PBMC in HCV patients with MC, one of the major B-cell disorders associated HCV infection, is significantly increased compared to HCV patients without MC or healthy controls (73). Interestingly, there was also a positive correlation between circulating gC1qR with RF activity and C1q concentrations in HCV patients with MC (73). Taken together, these observations suggest the involvement of gC1qR in the pathogenesis of HCV-induced autoimmunity.

## Role of C1q in HCV-Induced MC

Mixed cryoglobulins are cold-precipitable complexes of monoclonal or polyclonal IgM RF with polyclonal or oligoclonal IgG (15). Type II MCs, which are composed of monoclonal IgMκ RF and polyclonal IgG, are heavily represented among cryoglobulins associated with chronic HCV infection, and those found in patients with primary Sjogrens syndrome, both of which may be complicated by clonal B-cell proliferations and specific types of non-Hodgkin’s lymphoma (15). HCV patients with symptomatic type II MC suffer from various extrahepatic manifestations, including vascular, renal, and neurological lesions (74), i.e., cryoglobulinemic vasculitis (CryoVas) (75).

In type III MCs, both the IgM and IgG components appear to be polyclonal; extrahepatic disease manifestations may occur, but cryoglobulin levels are lower than type II, and an association with asymptomatic disease is more frequent. In addition, intermediate types characterized by oligoclonality or mixed IgM clonality with polyclonal IgM (type IIa) have also been described (76). Type III MCs may be found in HCV infection, as well as associated with rheumatic diseases [e.g., systemic lupus erythematosus (SLE)] in which complement activation may occur (16). The significance of Type IIa and related intermediate forms with regard to the progression to clonality that may occur in cirrhosis associated with HCV, and in primary Sjogrens syndrome, remains to be fully defined (77, 78).

As noted, a low serum level of C4 is a significant “signature” of type II MC patients (15, 16). This selective depression of C4 strongly implicates classical pathway activation of the complement in cryoglobulin formation. However, the level and function of C4 may be significantly influenced by inter-individual copy-number variation of C4A or C4B genes, charge variation, or isotype deficiency of these genes (79). Incorporation of C1q into isolated cryoprecipitates was first demonstrated as an 11S peak on density gradient ultracentrifugation in patients with lupus nephritis (80). More recent studies have confirmed that cryoprecipitates from patients with HCV-associated CryoVas are enriched in C1q (81), antibodies to HCV antigens (82), and may contain HCV-core protein as indicated by results obtained using an enhanced highly sensitive chemiluminescent microparticle immunoassay (81). Based on evidence that C1q and HCV-core bind to gC1qR, gC1qR/HCV-core complexes might provide a platform for complement activation and deposition of C4D at sites of vasculopathy (83). Additional factors that might be reflected in depletion of C4/C1q and localization to cryocomplexes include the ability of C1q to bind promiscuously to >100 known ligands, including both IgG- and IgM-containing immune complexes, surface-bound C-reactive protein, and molecules exposed at the surface of apoptotic cells, with binding through charged residues on the apex of the gC1q heterotrimer (19, 84), acquired C1-inhibitor deficiency (85), regulation of activation by C4-binding protein (86, 87), and antibodies to C1q and/or potentially to other components of the C1 complex (88).

## Role of RF in MC Pathogenesis

Although MC may be associated with a rheumatoid-like arthritis, it is distinct from RA in that antiglobulin activity is restricted to the IgM isotype; although it is presumed that the antigen specificity is directed primarily to determinants in the Fc portion of IgG uncovered by aggregation or complexing to antigen, there have been some suggestions of F(ab)′2 anti-hinge or anti-idiotypic specificity. Low-affinity RFs (Kd~10^−5^M) are natural antibodies cross reactive with other autoantigens, whereas high-affinity RFs (Kd~10^−7^M) have undergone affinity maturation and are hypermutated (89). The RF response is broadly represented in a number of infectious etiologies, either as a response to immune complexes formed by the infecting microbe and antibodies or as a function of direct infection and polyclonal B-cell stimulation. In Type II cryoglobulins, the contribution of IgM and IgM RF to total protein content presents a spectrum of concentrations and clonality, ranging from cryocomplexes with RF activity greatly exceeding serum levels and enhanced representation of IgM cross-reactive idiotypes (notably the Wa idiotype first described 45 years ago) (90). This allows for the observation of RF in mixed cryoglobulins in patients with apparently negative RFs, and mixed cryoglobulins that are composed almost entirely of IgM with kappa light chains. It has also been reported that HCV virion in fractionated mixed cryoglobulins is complexed with VLDL, providing a mechanism for localization of virus to sites of pathology *via* the LDL receptor (91), and the potential for LDL receptor genetic polymorphisms to influence disease outcome (92). RFs studied from B-cell clusters isolated by microdissection of liver biopsies from HCV-infected patients were hypermutated and overlapped in regard to variable region gene usage with blood and bone marrow (93).

## Apoptotic Role of C1q in SLE

Activation of complement is a central feature of SLE, intimately related to the pathogenesis of lupus nephritis, and a marker for disease activity and relapse; deficiencies or polymorphisms of molecules central to the classical, alternative, and lectin pathways have been linked to disease susceptibility, immune-complex nephritis, and severity (94). In particular, a central role for C1q and C1q receptors, both with regard to deficiency and as molecular cell-surface sensors for innate and acquired immune responses, has been reviewed (95). The observation that SLE develops in ~90% patients genetically deficient in C1q highlighted a function of C1q as “protector” against autoimmunity that may be independent of its classical role ion complement activation (96). In SLE, C1q deficiency may result either from genetic disorders or anti-C1q autoantibodies (97). The contributions of C1q deficiency in the development of SLE can be related to abrogation of binding to molecules (phosphatidylserine, double-stranded DNA, glyceraldehyde-3-posphate dehydrogenase, annexins 2 and 5, calreticulin) expressed on the surface of dying cells (17) and resultant lack of generation of activated C1s to cleave these apoptotic autoantigens (19). In addition, all three pathways of complement can be activated on the surface of apoptotic cells without further activation of innate or adaptive immune components (17); impaired clearance of dying cells and immune complexes in absolute or functional C1q deficiency is linked to the development of self-reactive B cells with affinity toward multiple autoantigens, and effects on monocyte and dendritic cell differentiation (98, 99).

Anti-C1q autoantibodies have been reported in 30–50% of SLE patients, most commonly correlating with antibodies to double-stranded DNA, nephritis, and low levels of C3 and C4 (100, 101). While antibodies with unique specificities for the globular head and collagen tail of C1q have been identified, the impact of blocking C1q domains on biological activity remains uncertain compared to a number of other (e.g., calreticulin) known inhibitors (17). Although the C1q is a major component of HCV-induced cryoprecipitate, to the best of our knowledge there are no published studies addressing this issue. Low levels of C1q and a significant prevalence of anti-C1q autoantibodies are shared features of SLE and HCV-induced cryoglobulinemia (36, 73, 102, 103).

Interferon-alpha is well-known to have both antiviral and inflammatory effects (104). Plasmacytoid dendritic cells (pCD) are the major producers of IFNα (105). Interestingly, C1q collagen tail interacts with LAIR-1 (CD305), an inhibitory receptor for C1q (106), on pCDs and restricts the production of IFNα (107). Therefore, anti-C1q autoantibodies might contribute to HCV-induced cryoglobulinemia by blocking the interaction between the C1q tail and its inhibitory receptor, LAIR-1, on pDCs resulting in uncontrolled overproduction of IFNα, which may in turn drive the inflammation associated with progression of MC in HCV patients. Alternatively, elevated levels of IFNα produced by uncontrolled pCDs might promote differentiation of B cells into plasma cells resulting in production of pathogenic autoantibodies reported in SLE (108).

## Conclusion

The complement system plays a central role in rheumatic and autoimmune diseases, several of which are associated with the presence in blood of cold-perceptible immune complexes enriched in IgM RF, specific antibody activities, putative antigens and C1q as part of a cascade capable of activating the classical pathway, leading in turn to the generation of anaphylatoxins, chemotactic factors, and inflammatory mediators. Both C1q and its globular receptor are promiscuous with regard to ligand specificity, allowing for alternative functions that include binding to specific intracellular antigens expressed on the surface of apoptotic cells, as well as to specific domains of HCV. A research agenda includes the mapping of C1q epitopes responsible for binding to diverse ligands, anti-C1q antibodies, heterotrimeric formation, and C4/C2 serine protease generation that might in turn be targets for therapy. Similar therapeutic strategies might be targeted to gC1qR binding to C1q and High Molecular Weight Kininogen in plasma, on the surface of endothelial cells as a mechanism for vasculopathy, and the regulation of danger sensors on mononuclear cells and immature dendritic cells. A second line of investigation is the delineation of factors responsible for the strikingly low C4 levels in sera of patients with Type II MC and some patients with SLE with regard to mechanisms such as copy-number variation, polymorphisms, cleavage and deposition in tissue, and specific inhibitors. With regard to HCV, a focus on liver pathology would provide an arena to identify complement-defined mechanisms of disease, including immune activation in lymphoid follicles, steatosis, fibrosis, and regeneration.

## Author Contributions

AE and PG wrote the review. AB and TS revised the manuscript.

## Conflict of Interest Statement

The authors declare that the research was conducted in the absence of any commercial or financial relationships that could be construed as a potential conflict of interest.
